# Patterns and correlates of objectively measured physical activity in 3-year-old children

**DOI:** 10.1186/s12887-020-02100-1

**Published:** 2020-05-12

**Authors:** Linnea Bergqvist-Norén, Elin Johansson, Lijuan Xiu, Emilia Hagman, Claude Marcus, Maria Hagströmer

**Affiliations:** 1 Department of Clinical Science, Intervention and Technology, Karolinska Institutet, Blickagången 6A, S-141 57 Stockholm, Huddinge Sweden; 2 Department of Neurobiology, Care Sciences and Society, Karolinska Institutet, S-141 83 Stockholm, Sweden; 3 Allied Health Professional Function, Karolinska University Hospital, S-141 86 Stockholm, Sweden; 4 Department of Health Promoting Science, Sophiahemmet University, S-114 86 Stockholm, Sweden

**Keywords:** Accelerometer, ACTIGRAPH GT3X +, Counts per minute (CPM), Vector magnitude, Childhood obesity, Preschoolers, Socioeconomic status

## Abstract

**Background:**

To increase the knowledge about physical activity (PA) patterns and correlates among children under the age of 4, there is a need for study’s using objective measurements. The aim of this study was therefore to investigate if objectively measured PA among 3-year-old children differed between day of week and time of day and whether it correlated to child weight status and sex as well as parental weight status and education.

**Methods:**

Totally 61 children (51% girls) aged 3, participating in Early Stockholm Obesity Prevention Project were included. PA was measured with a tri-axial accelerometer (ActiGraph GT3X+) worn on the non-dominant wrist for one week. The main outcome was average PA expressed as counts per minute from the vector magnitude. PA and demographics/family-related factors were collected at baseline and at age 3. To analyze the results simple linear regression, ANOVA and paired t-tests were performed.

**Results:**

The mean number of valid days was 6.7 per child. The children were more active on weekdays than weekends (*p* < 0.01) and the hourly pattern differed over the day with children being most active midmorning and midafternoon (*p* = 0.0001). Children to parents with low education were more active (*p* = 0.01) than those with highly educated parents. No differences in PA by child weight status, sex nor parental weight status were found.

**Conclusions:**

PA in 3-year-old children was lower during weekends than weekdays and varied over the day. Boys and girls had similar PA patterns, these patterns were independent of child or parental weight status. Children to parents with low education were more active than their counterparts. The fact that PA differed between weekdays and weekends indicates that PA might be affectable in 3-year-old children.

## Background

Being physically active is essential for reducing the risk of premature death, cardiovascular disease, cancer, diabetes, chronic respiratory diseases and mental illness in the adult population [1, 2]. Even among children and adolescents there are positive health effects of PA, including cardiorespiratory and muscular fitness, bone health and weight status/adiposity [3]. In preschoolers, there is a positive association between PA and bone health as well as a reduced risk for excessive increase in body weight and adiposity [3]. For this reason, it is of value to ascertain why some individuals are active and others not and what factors that correlates to an active lifestyle. The gained information might improve the development of more effective PA interventions which aims to increase PA.

Objective measurements with motion sensors/accelerometers have made it possible to study patterns of PA over a period, e.g. variations over the day or between days. Previously, studies have found indications that children are less active during weekends than weekdays [4–7]. This type of information can help form PA interventions in a more beneficial way, meaning to focus on increasing activity when children might already be less active, that is on weekends. However, if PA differs among 3-year-old children between day of week have yet to be established and in the literature, there has been diverse results [8].

Studies on children > 4 years and adolescents have, for instance, identified the following correlations to PA, age; PA decreases yearly after the age of 5 [7–9], sex; boys are more active and less sedentary than girls [7–9], and weight status; normal weight children seems to be more active than overweight and/or obese children [9–14]. The association between Socioeconomic status (SES) and PA has been studied in older children’s (age ≥ 6) PA, but results are inconsistent. Some found children from low SES to be more physically active, but others the opposite or no correlation at all [8, 9, 15–18]. However, these correlates have not been determined at the age of 3 and it has been suggested that more research, using objective data, is needed in this age-group [8]. Further, parental obesity has been established as a predominant risk factor for childhood obesity [19, 20], but whether parental obesity predicts PA among preschool children is unknown.

Given the knowledge gap of factors and patterns correlated to PA in children below the age of 4, we aim to investigate the patterns of PA over the course of the day as well as over the week. Further we aim to investigate whether PA correlates to child weight status and sex as well as parental weight status and education among 3-year-old children within the Early Stockholm Obesity Prevention Project (Early STOPP).

## Methods

### Participants

Early STOPP is a longitudinal clustered randomized controlled trial (RCT) with an obesity prevention intervention including 238 children with a 5-year consecutive follow-up period. The children have high (*n* = 181) or low (*n* = 57) risk of developing obesity based on parental BMI; where high-risk is defined as 1 parent with obesity (BMI ≥ 30) or 2 parents with overweight (BMI 25–29.9). Low-risk is defined as having both parents with BMI < 25 [21]. The families were recruited when the child was 1 year old from Child Health Care Centers (CHCC) in the Stockholm region between fall 2009 and spring 2013. The high-risk families were randomized to intervention (*n* = 66) or control group (*n* = 115), based on the CHCCs (cluster) and low-risk families serves as a reference group. More information about the project and the intervention can be found in earlier published papers [21–23]. To reduce a potential effect of the intervention, the intervention group was excluded in the present study. Among families in the control and low-risk groups (*n* = 172), 100 families attended the annual visit at 3 years of age (second wave follow-up), taken place ±2 months from the child’s third birthday.

Early STOPP was approved by the Stockholm regional ethics committee in Stockholm in March 2009 (file no. 2009/217–31). The parents signed a written consent to be a part of the Early STOPP project and this sub-study falls under the project’s intent.

### Physical activity

PA was assessed using a tri-axial motion sensor, the Actigraph GT3X+ accelerometer (Actigraph, Pensacola, FL). Children wore the accelerometer on their non-dominant wrist, wrist dominance estimated by the parents, for seven consecutive days. For a day to be considered valid it had to contain at least 10 h of PA measurements [24, 25] and all children with less than 4 valid days, or missing weekend data, were excluded [26]. The data was collected between June 2012 and June 2015.

Data was analyzed in the ActiLife program, version 6.11.9. (Actigraph, Pensacola, FL). Night-time sleep was excluded by removing the hours between 8.45 pm – 7.20 am [27] and any potential daytime sleep was considered as sedentary time. The outcome variable was average PA expressed in counts per minute (CPM), with a sampling rate of 30 Hz [28]. We used CPM from the vector magnitude (VM) a variable that combines the 3 axes in 1 outcome defined as √(x^2^ + y^2^ + z^2^). The outcome was calculated for every hour and for weekdays and weekends respectively.

### Weight status

Body weight was measured to the nearest 0.1 kg with a portable scale Tanita HD-316 (Tanita corp, Tokyo, Japan) and height to the nearest 0.1 cm with a fixed stadiometer (Ulmer; Buss Design Engineering, Elchinge, Germany). BMI (kg/m^2^) was calculated for children at age 3, second wave follow-up, classifying children as normal weight, overweight or obese using international cut-off values corresponding to adult BMI 25 and 30 [29]. Parental BMI was calculated at baseline and at second wave follow-up using international standards to classify normal weight (BMI 18.5–24.9), overweight (BMI 25–29.9) and obesity (BMI ≥ 30).

### Demographics and family-related factors

Parental educational level was used to determine SES [30]. Mothers and fathers reported their highest level of education as; Elementary school, High school or Academic education. Parental education level was considered high if at least 1 parent had an academic education, otherwise as low [22, 23].

Additional demographic and family-related information was collected in order to adjust for possible confounders [31]. Child daycare was reported by the parents as either staying at home with a parent and/or guardian or preschool care. Preschool was then categorized as full time (≥ 30 h/week) or part time (< 30 h/week). Country of origin was categorized as either Nordic or non-Nordic, where non-Nordic was defined as at least 1 parent born in a non-Nordic country. Number of siblings were collected as either 0, 1 or ≥ 2. Season of measurement was categorized as spring (March–May), summer (June–August), fall (September–November) and winter (December–February). All information was derived from questionnaires filled out by the parents in connection to the visit.

### Statistical analysis

Data were presented as means and standard deviations (SD) or frequency (n) and percentage (%), depending on their natures. To compare compliers and non-compliers to the inclusion criteria for the accelerometer, a response analysis was performed using Pearson’s Chi-Square tests and independent *t*-tests on descriptive variables (sex, weight status, age and parental education).

Factorial repeated analysis of variance (ANOVA) was performed in order to evaluate differences between days of week and time of day for the main outcome CPM. A paired t-test was performed to compare differences in PA between weekdays and weekends. To test if PA differed (values through all days/weekdays/weekend days) by child weight status and sex and parental weight status and education, independent ANOVA, with a post hoc equation Bonferroni, was performed. Significant factors (*p* < 0.05) were further included in a model using simple linear regression, adjusting for: sex, age, family group, daycare, country of origin, number of siblings and season [31]. Cohen’s d were used to calculate effect size for group comparisons, with cutoffs 0.2, 0.5 and 0.8 indicating a small, medium or large effect size respectively [32]. Post hoc power equation for multiple regression was used to calculate power. All analyses were performed with IBM SPSS Statistics version 23.0 (IBM, Armonk, New York, USA).

## Results

**Fig. 1:**
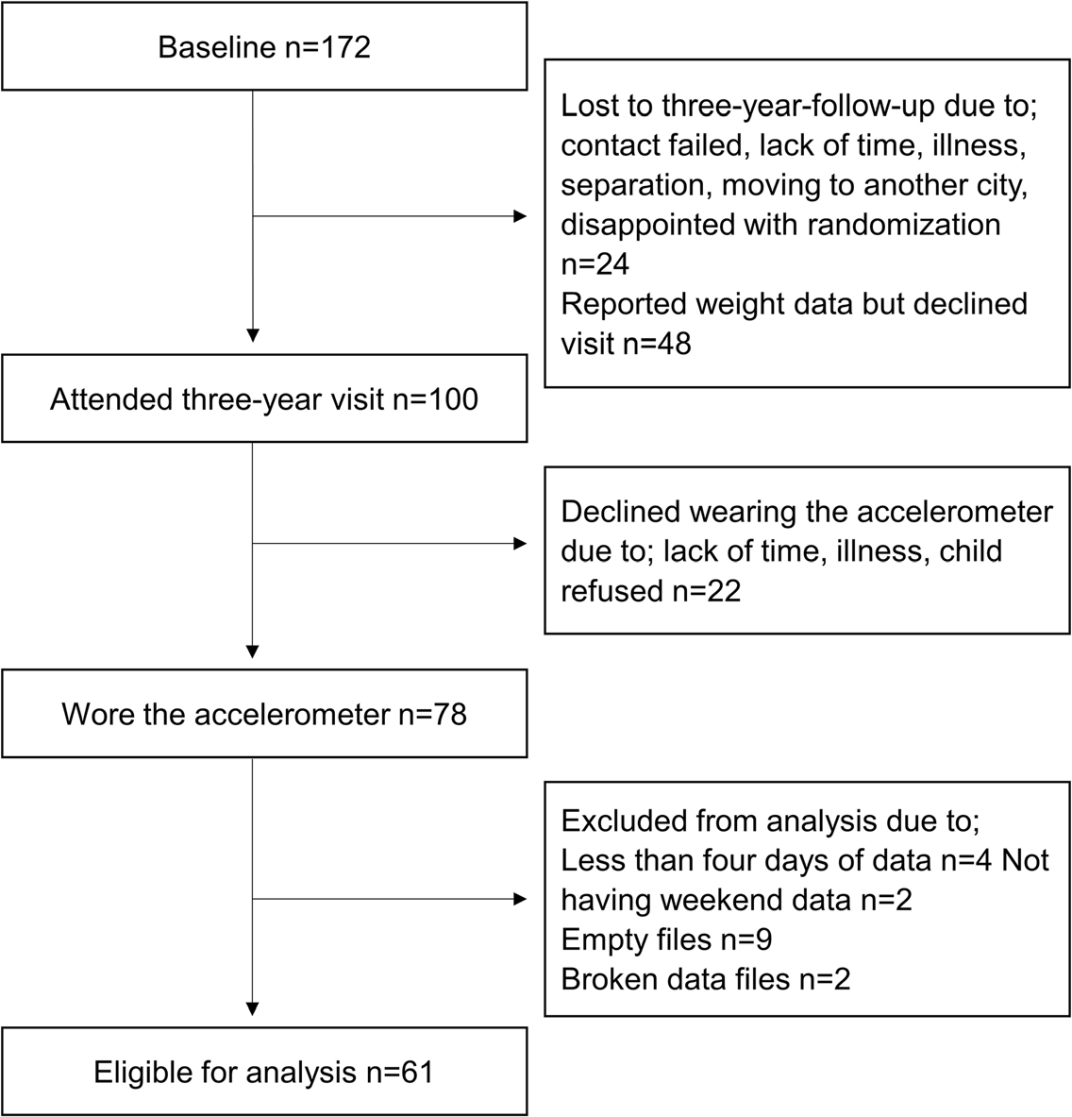
Flowchart of participants from the Early STOPP cohort eligible for analysis in the present study

In total 61 families were included in the current analysis. No differences were found between included and excluded participants with respect to sex, weight status, age or parental education (data not shown). A flowchart of the participants is presented in Fig. 1, and descriptions of participant characteristics in Table 1. There were 9 children with overweight and 1 with obesity, these subjects were the merged to create 1 group. Only 2 children were not participating in preschool care, hence this group was pooled with the group of children spending less than 30 h/week in preschool care. In total 82% of the families had high educational level and 85% were of Nordic country background. The mean number of valid days of accelerometer data was 6.7 days (0.6 SD) per child.

**Table 1:** Sociodemographic and anthropometric characteristics of study participants (*n* = 61)

	Mean ± SD	N (%)	Missing n (%)
**Age**	3.06 ± 0.08		
**Sex**			
Girls		31 (50.8)	
Boys		30 (49.2)	
**Family group** (a)			
High risk		41 (67.2)	
Low risk		20 (32.8)	
**Child weight status**			1 (1.8)
BMI (kg/m^2^)	16.7 ± 1.2		
Normal weight		50 (83.3)	
Overweight		9 (15.0)	
Obesity		1 (1.7)	
**Parental weight status** (b)			
*Mother*			3 (4.9)
BMI (kg/m^2^)	28.3 ± 5.4		
Normal weight		18 (31.0)	
Overweight		17 (29.3)	
Obesity		23 (39.7)	
*Father*			5^¤^ (8.2)
BMI (kg/m^2^)	26.8 ± 6.5		
Normal weight		25 (44.6)	
Overweight		17 (30.4)	
Obesity		14 (25.0)	
**Family Education** (c)			1 (1.6)
Low		11 (18.3)	
High		49 (81.7)	
**Childcare** (d)			8 (13.1)
Preschool full-time		34 (64.2)	
Preschool part-time		18 (34.0)	
Other		1 (1.9)	
**Country of origin**			9 (14.8)
Nordic		44 (84.6)	
Non-Nordic		8 (15.4)	
**Siblings**			8 (13.1)
0		7 (13.2)	
1		30 (56.6)	
≥ 2		16 (30.2)	
**Season for measurement**			
Spring		6 (9.8)	
Summer		9 (14.8)	
Fall		29 (47.5)	
Winter		17 (27.9)	

*Abbreviations*: *BMI* Body Mass Index, *SD* Standard deviation

(a) Family group based on parental BMI High: 2 parents with BMI ≥25 or 1 parent with BMI ≥30 and low: both parents with BMI ≤ 25

(b) Weight status: BMI categories for adults and corresponding categories for children according to Cole et al.

(c) Family education definition: Low: neither parents have an academic education High: at least 1 parent have academic edu

(d) Child care, full time ≥ 30 h/day part time < 30 h/day

^¤^Besides missing data due to father did not show or communication failed this also include father unknown

**Fig. 2:**
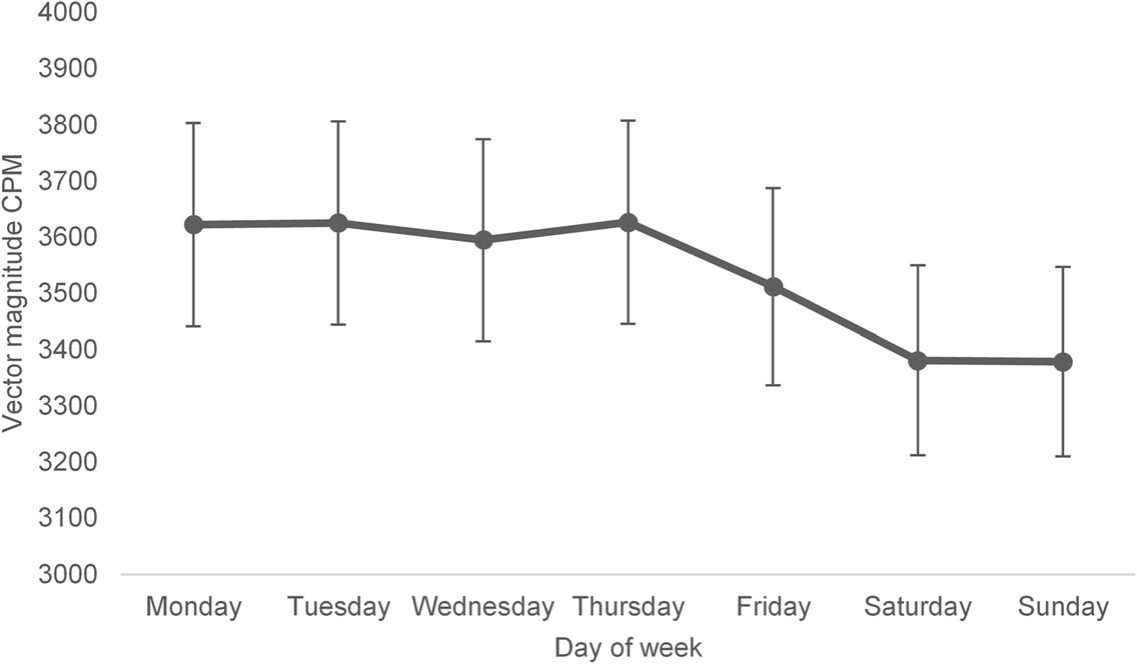
The weekly average physical activity, mean vector magnitude CPM (95% CI). CPM = Counts per minute

**Fig. 3:**
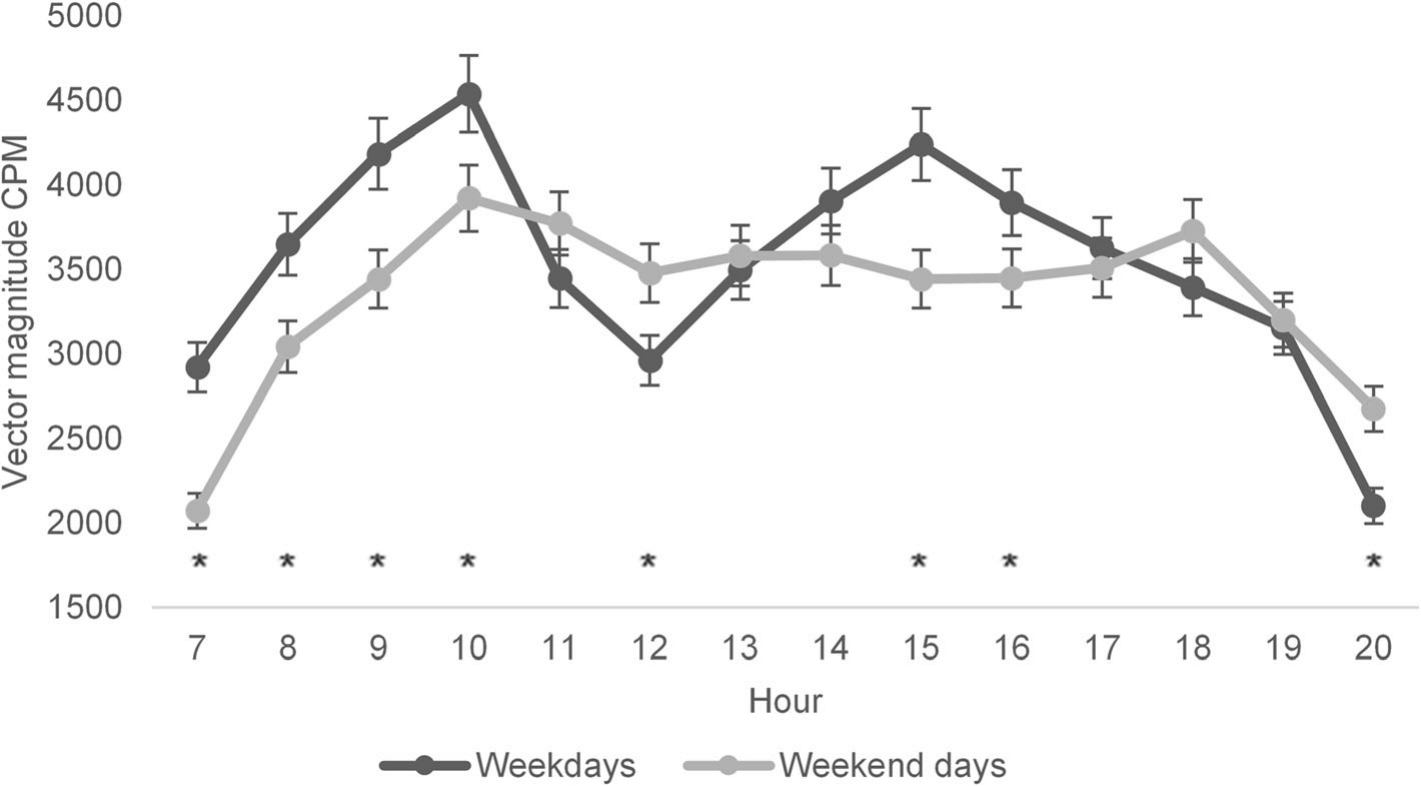
The hourly average physical activity on weekdays and weekends, mean vector magnitude CPM (95% CI). * = *p* - value < 0.05 for differences between weekdays and weekends. CPM = Counts per minute

There were no differences between weekdays in average CPM (Fig. 2). However, children were more active during weekdays than weekends (*p* = 0.01) with Cohen’s d 0.34, as provided in Table 2. The factorial repeated measures ANOVA between hours per day showed differences depending on time during the day (*p* < 0.0001), with children being most active mid forenoon (around 10 am) together with midafternoon (around 3 pm) (Fig. 3). Moreover, when comparing the weekday hourly pattern to the weekend hourly pattern PA differed on the hours 7-10 am, 12 pm, 3–4 pm and 8 pm (Fig. 3). On the hours 7–10 am and 3–4 pm children were more active on weekdays, however on the hours 12 pm and 8 pm the activity was higher on weekends.

**Table 2:** Weekdays and weekend differences in average physical activity

	Weekdays	Weekend days	*P*-value	Cohen’s d (a)
	Mean VMCPM ± SD	Mean VMCPM ± SD		
**Total sample**	3587 ± 624	3392 ± 695	0.01*	0.34

*Abbreviations*: *SD* Standard deviation, *VMCPM* vector magnitude counts per minute

(a) Cohen’s d = effect size

*= *p* < 0.05

There were no differences in PA regarding child weight status and sex (Table 3). Furthermore, no differences in PA comparing the high-risk families to the low-risk families, nor the parental weight status at second wave follow-up were found (Table 3). There was a difference in activity based on family education on both total activity of the week (*p* = 0.03 Cohen’s d 0.7) and on weekdays (*p* = 0.01, Cohen’s d 0.8). These differences remained significant for weekdays in the adjusted analysis (*p* = 0.02) but not for the total activity (*p* = 0.05) (Table 3). None of the other factors (child daycare, country of origin, number of siblings and season) showed any association to the children’s PA (data not shown).

**Table 3:** Physical activity by child weight status, sex, family group, parental weight status and family education

Variables	All days	Weekdays	Weekends
	Mean VMCPM ± SD	Mean VMCPM ± SD	Mean VMCPM ± SD
**Total sample***n* = 61	3538 ± 570	3598 ± 615	3374 ± 710
**Child weight status** (a)			
Normal weight *n* = 50	3513 ± 589	3579 ± 645	3330 ± 699
Overweight *n* = 10	3662 ± 507	3706 ± 494	3553 ± 800
*P*	0.5	0.6	0.4
**Child sex**			
Boys *n* = 30	3518 ± 637	3564 ± 659	3402 ± 793
Girls *n* = 31	3557 ± 507	3630 ± 589	3347 ± 632
*P*	0.8	0.7	0.8
**Family group** (b)			
Low *n* = 20	3417 ± 427	3442 ± 463	3366 ± 491
High *n* = 41	3595 ± 625	3673 ± 669	3378 ± 801
*P*	0.3	0.2	0.9
**Maternal weight status** (c)			
Normal weight *n* = 18	3375 ± 474	3394 ± 485	3337 ± 633
Overweight *n* = 17	3451 ± 598	3523 ± 660	3247 ± 731
Obesity *n* = 23	3699 ± 574	3754 ± 639	3536 ± 786
*P*	0.2	0.2	0.4
**Paternal weight status**			
Normal weight *n* = 25	3479 ± 503	3579 ± 587	3220 ± 582
Overweight *n* = 17	3688 ± 743	3707 ± 737	3659 ± 860
Obesity *n* = 14	3478 ± 476	3464 ± 496	3507 ± 598
*P*	0.5	0.6	0.1
**Family education** (d)			
Low *n* = 11	3864 ± 599	4003 ± 615	3470 ± 910
High *n* = 49	3457 ± 546	3498 ± 586	3347 ± 674
*P*	0.03*	0.01*	0.7
**Family education-**			
**Adjusted p-value** (e)	0.05	0.02*	

*Abbreviations*: *SD* Standard deviation, *VMCPM* vector magnitude counts per minute

(a) Body mass index (BMI) categories for children according to Cole et al.

(b) Family group based on parental BMI; High: 2 parents with BMI ≥25 or 1 parent with BMI ≥30 and low: both parents with BMI ≤ 25

(c) Weight status according to BMI categories as following; normal weight 18.5–24.9, overweight 25.0–29.9 and obesity > 30

(d) Family education definition: Low: neither parents have an academic education High: at least 1 parent have academic education

(e) adjusted for; Age, sex, family group, preschool care, parental country of origin, number of siblings and current season

*= *p* < 0.05

## Discussion

### Main results

This cross-sectional study contributes to the knowledge regarding young children’s PA patterns and its correlates. Our findings herein are an extension of our previous findings on 2-year-old children [22], that young children vary their activity pattern over the days as well as between weekdays and weekends. Furthermore, child PA was inversely associated to parental education with a large effect size. No differences in PA between boys and girls was found.

In the present study, children were less active during weekends than weekdays which is in line with previous findings [4–7, 22, 33]. During weekends children are primarily in care of their parents/guardian and one hypothesis may be that parents do not have the same time to activate their children or that they consider PA as something the preschools should provide [33]. It has also been suggested that higher PA during weekdays could be due to the planned time for outdoor activities in preschools [24, 34]. The hourly PA-pattern differed between weekdays and weekends indicating that the variability could be caused by exogenous factors and that it might then be possible to influence the PA. The differences at 7 am and 8 pm might be explained by the fact that children sleep in during weekends and at the same time also stay up later at night [35]. The hourly pattern most likely confirms that preschools, in greater extent than parents, engage children in activities that are more physically active and have a fixed schedule for meals and naptimes. To further examine the parent-child PA relationship a comparison between their PA levels and patterns should be investigated in future studies.

We did not find any effect of weight status on PA. Studies on school-aged children, adolescents and adults have found normal weight individuals to be more active and less sedentary than individuals with overweight and/or obesity [9–14]. In preschoolers, there is a positive association between PA and a reduced risk for excessive increase in body weight and adiposity [3]. However, to our knowledge, weight status has, at 3 years of age, not been established as either a determinant of PA nor an outcome of too little PA [8].

Boys and girls were found to be equally active. In a review by Sallis et al. [9], 80% of the studies reported boys to be more physically active than girls among children age 4–17. Wijtzes et al. [31] reported sex-differences only on sedentary behavior but not on low PA or high PA in the same age group. Johansson et al. [22] reported no differences between boys and girls aged 2 within the Early STOPP population. Analyzing and exploring PA within this cohort should for this reason continue, in order to establish if, and in that case when, differences in PA by sex starts. Why boys are being more active than girls has yet to be established. Many factors have been suggested; motor skills, biological age, physical maturity, participation in sports but also social and cultural norm behaviors [36–42]. Most of these proposed factors applies primarily to older children however, motor skills could be a factor of interest for children younger than 4 years of age and should be considered to include in future studies.

Finding and targeting groups of individuals with risk for low PA might help improve effects of PA interventions [1]. As the predominant risk for childhood obesity [19, 20] parental weight could therefore be a target of interest. Meaning, if parental weight status correlates to children’s PA, children to parents with obesity might benefit from PA-interventions and the added knowledge may make interventions more efficient. However, in this study we could not find differences in PA by parental weight status and to the best of our knowledge no other study has investigated this in 3-year-old children. Previously Johansson et al. [22] found no differences in PA among 2-year-old children with respect to parental weight.

We found that children to parents with a lower educational level were more active on weekdays than their counterparts, with a large effect size. Previous results have been inconsistent and contradictory. In children from 6 years of age SES has in some studies been associated with PA [7, 8, 15–18]. A systematic review showed that 58% of the included studies reported a positive relationship between SES and PA and the remaining reported the opposite or no relationship [17]. The authors argue that studying this relationship is problematic due to the different types of measurements on both variables [17, 30, 43] that is, subjective or objective PA [17, 43] and various proxy for SES, for instance education, occupation and income [30, 44]. This could lead to the contradictory results of the studies and could therefore make it somewhat hard to compare findings [15, 17]. Even so, Beckvid Henrikson et al. [15] also found that six-year-old children from families with low SES were more active and less sedentary, using objective PA and education as a proxy for SES [15]. They discuss the possible implication of age on the differences in results, arguing that at an older age spontaneous PA is replaced with more structured sports [15, 16]. Resulting in the strengthening of a positive relationship to SES, since the coast for activity increases [15, 16]. Our results on 3-year-old children might cohere with their hypothesis that the correlation between SES and PA might differ depending on age.

### Strengths and limitations

We used an accelerometer (Actigraph GT3X+) to objectively measure PA with a mean of 6.7 valid days, providing robust data [25, 26, 45, 46] making us able to provide a picture of the children’s PA pattern hour by hour. Accelerometry is the suggested and most preferable method to be used when measuring younger children [45]. Although wrist placement is not the most common wear placement it has been shown to increase the wear compliance among children [46]. Using a uniform sleep-time for all children on both weekends and weekdays may be a source of uncertainty in the interpretation of the results. Nevertheless, children at this age often have regular hours independent of day of week [35].

Due to the relatively small sample from the Stockholm area, the higher proportions of highly educated parents with mostly Nordic country background compared to the Swedish population [47], the results should be interpreted with caution to other populations. Moreover, in this study we did not have information on motor skills or parental PA that might be of value for the total PA in this age group. Education as proxy for SES is the most commonly used, but using only 1 variable for a person’s SES is questionable and creating an index might give a more equitable estimation [30]. However, there are no agreed upon definition on what variables to then include [30].

## Conclusion

PA in 3-year-old children was lower during weekends than weekdays and varied over the day. Boys and girls had similar PA patterns, and these patterns were independent of child or parental weight status. Children to parents with a low educational level were more active than their counterparts. The fact that the PA differed between weekdays and weekends indicates that PA might be affectable in 3-year-old children.

## Data Availability

Data can indirectly be traced back to the study participants, and according to Swedish and EU personal data legislation this means that access can only be made upon request. The request should in this case be addressed to the PI Claude Marcus, and will be handled on a case by case basis. Any sharing of data will be regulated via a data transfer and use agreement with the recipient.

## Abbreviations

BMI: Body mass index
CPM: Counts per minute
PA: Physical activity
SES: Socioeconomic status
